# Supplementary material for: A Comprehensive Technology Platform for the Rapid Discovery of Peptide Inhibitors against SARS-CoV-2 Pseudovirus Infection
Source: Int J Mol Sci. 2023 Jul 29;24(15):12146. doi: 10.3390/ijms241512146 (PMC10418426; doi:10.3390/ijms241512146)
Supplement: Supplementary file 1 [file ijms-24-12146-s001.zip › ijms-2468763-supplementary.pdf]

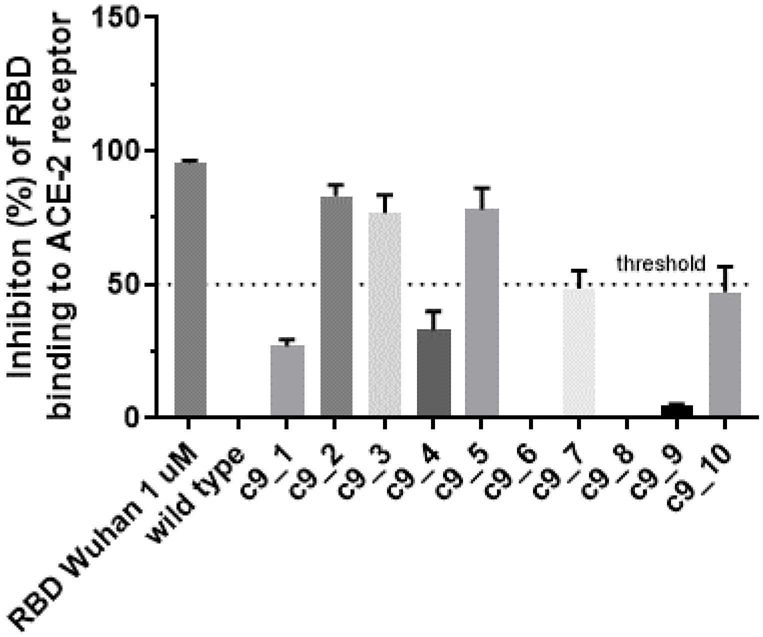

Supplementary Figure S1: Inhibition of RBD-ACE2 interaction by cyclizable peptide c9 variants

Inhibition of RBD-Fc (Wuhan) binding to ACE2 by 100 µM cyclizable peptide variants (c9 wild type, c9_01 to c9_10) and the 1 µM RBD (non-tagged Wuhan variant) protein serves as a positive control. The peptides and the positive control were pre-incubated with ACE2 before the addition of the RBD-Fc detection agent from the ELISA kit. The broken line indicates the threshold of greater than 50% inhibition, where peptides

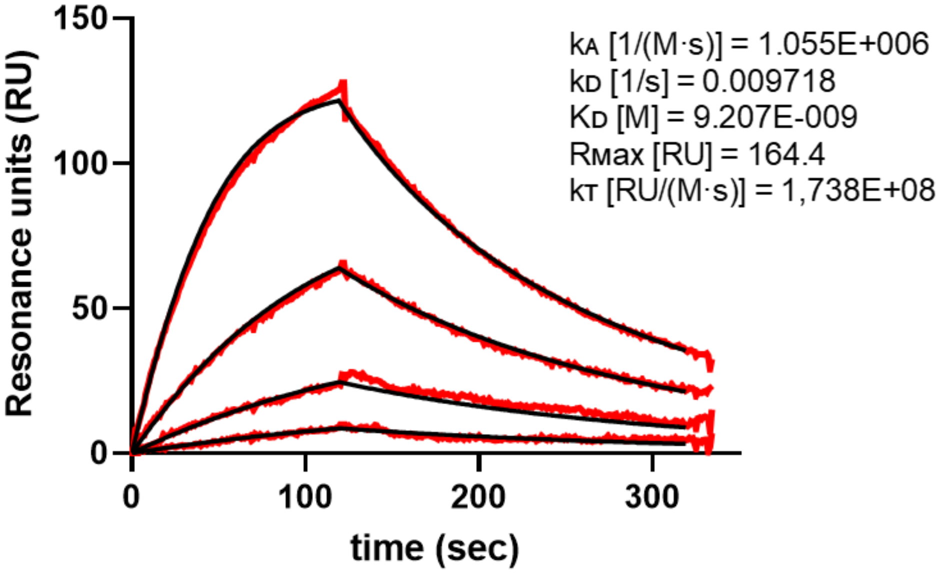

are considered active.

Supplementary Figure S2: **Real-time monitoring of Wuhan type RBD binding to ACE2 using SPR.**

Sensorgrams at different concentrations (1, 3, 10, and 30 nM) of RBD Wuhan type in PBST (red curves) binding to ACE2 immobilized on the sensor chip. The fitted curves (black) using the Langmuir model with mass transport are shown. Inset displays the fitting parameters obtained from the analysis.

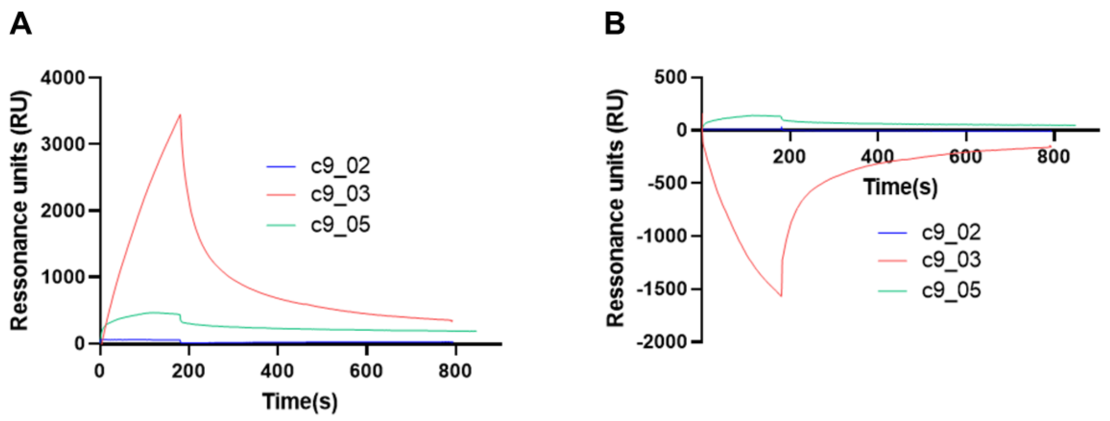

Supplementary Figure S3: Assessment of Non-Specific Binding for c9_02, c9_03, and c9_05 in SPR Experiment.

Non-specific binding of c9_02, c9_03, and c9_05 in SPR experiment A) Raw sensorgrams of c9_02, c9_03, and c9_05 at the highest concentration (300 µM) on the reference channel and ACE2 channel. B) Difference spectra of c9_02, c9_03, and c9_05. The difference spectra were obtained by subtracting the reference sensorgram from the ACE2 sensorgram.

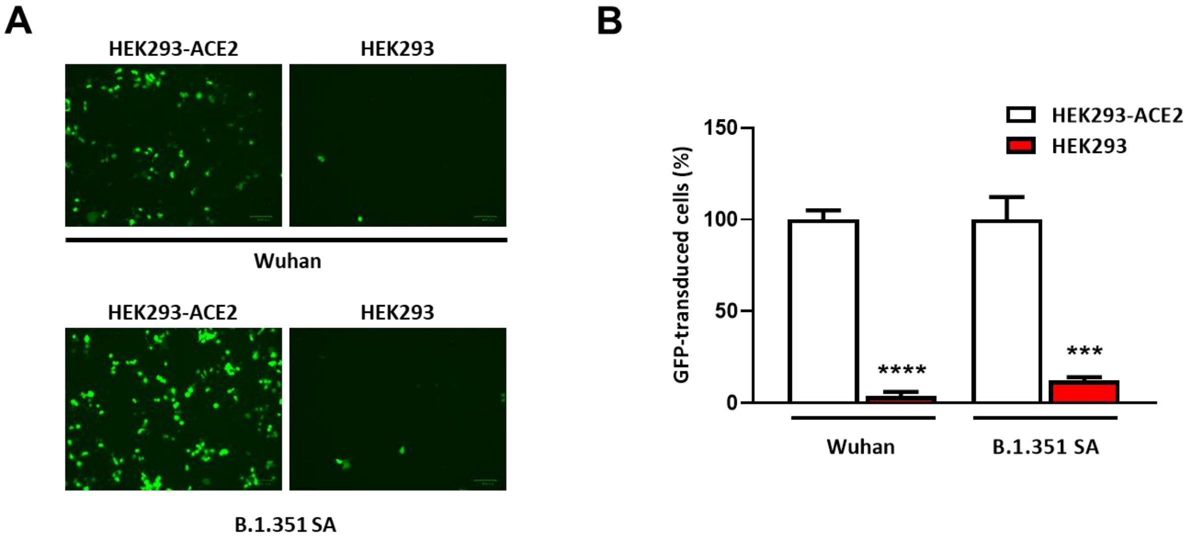

Supplementary Figure S4: Pseudoviral infectivity is mediated by SARS-CoV-2 Spike/ACE2 receptor interaction. (A, B) Transduction efficiency inhibition of pseudovirus exposing SARS-CoV-2 Wuhan or B.1.351 SA Spike variants in HEK293 compared to HEK293 stably expressing ACE2 receptor (HEK293-ACE2). *** $p<0.005$ and **** $p < 0.001$, Unpaired t-test.

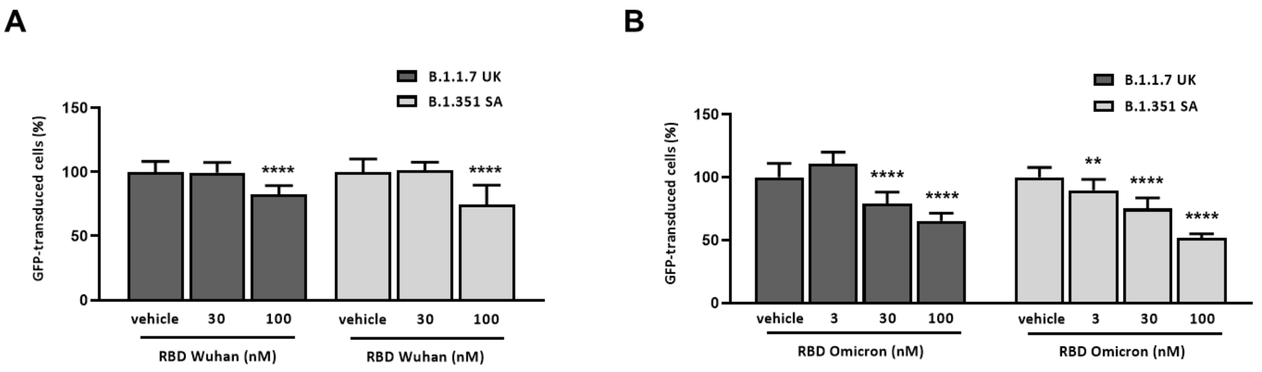

Supplementary Figure S5: RBD reduced pseudoviral transduction. Dose-response effect of RBD variants A) Wuhan or B) Omicron on the transduction of pseudovirus exposing SARS-CoV-2 B.1.1.7 UK or B.1.351 SA Spike variants in HEK293-ACE2 cells. ** $p < 0.005$ *vs* Vehicle, **** $p < 0.001$ *vs* Vehicle, one-way ANOVA, and Bonferroni *post hoc* analysis.

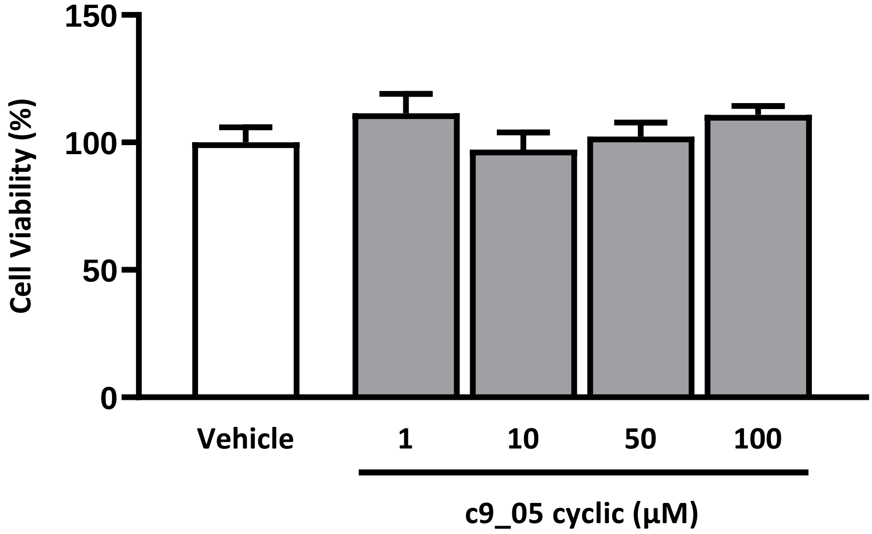

Supplementary Figure S6: The treatment with cyclic c9_05 did not affect HEK293-ACE2 viability. Data are the mean ± SD percentage of viable HEK293-ACE2 compared to vehicle-treated control cells.
